# Mushroom DNA barcoding project: Sequencing a segment of the 28S rRNA gene

**DOI:** 10.1002/bmb.21388

**Published:** 2020-06-25

**Authors:** Ivo R. Horn, Peter A. Verleg, Nafiesa Z. Ibrahim, Khadiedjah Soeleman, Floris van Kampen, Mia O. Ruesen, Naïsha M. Reulen, Henk Breij, Roderick J. Bakker, Barbara Gravendeel

**Affiliations:** ^1^ University of Applied Sciences Leiden, Faculty of Science and Technology Leiden The Netherlands; ^2^ Naturalis Biodiversity Center, Endless Forms Group Leiden The Netherlands; ^3^ Leiden Centre for Applied Bioscience Leiden The Netherlands; ^4^ Institute of Biology Leiden, Leiden University Leiden The Netherlands

**Keywords:** DNA barcoding, fungal identification, PCR, sanger sequencing

## Abstract

DNA barcoding is an important molecular methodology for species identification that was developed over the last two decades and it should be covered in the biology bachelor curriculum. Here, we present an example of DNA barcoding by sequencing a segment of the 28S nuclear ribosomal large subunit rRNA gene of wild mushrooms and framing the education in a project form for undergraduate students in biology. Students perform this project in 6–8 weeks, which also includes preparing a poster, writing a report and presenting a paper related to the work in a journal club format. First, fieldwork in the Netherlands was carried out, during which students collected mushrooms under supervision of a professional mycologist with the goal to (a) verify morphologically based identifications with a molecular method and (b) assess phylogenetic relationships of the different species collected. Next, DNA extractions and quantitation were performed, PCR amplification was done, and samples were sent out for Sanger sequencing. Students aligned and analyzed the sequences using BLAST and Geneious and subsequently created a phylogenetic tree. In case of collecting DNA barcodes of an earlier sequenced species, students could upload the data to a repository established for facilitation of future research projects. The method described is very robust, reagents and equipment are readily available, and costs are relatively low. In addition, the results can be compared to published fungal phylogenetic trees.

## 1 INTRODUCTION

DNA barcoding is a molecular methodology that identifies species using short genetic markers. It was first developed by Paul Hebert in 2003 for butterflies, and in 2008, a consortium of institutions joined forces under the name iBOL to start the ambitious task of building reference libraries for barcodes of all life on earth.^1^, ^2^ To identify mushrooms, DNA barcoding is seen as one of the most powerful tools since identification based on morphology is not always sufficient.^3^ To discriminate species, the nuclear internal transcribed spacer (nrITS) and the 28S nuclear ribosomal large subunit (LSU) rRNA marker sequences are generally used.^4^, ^5^, ^6^ The nrITS region of the rRNA gene cluster is the most commonly used target to identify fungi, which comprises a region of 600 bp. The major advantage of nrITS barcoding is the use of well‐validated primer sequences, detectability due to the large number of copies of the rRNA clusters and appropriate sequence variation in the nrITS genes between related organisms.^7^ However, recent research has shown that sequencing only the nrITS marker is not always sufficient for accurate species identification and therefore a combination of DNA sequencing of different markers is advised.^8^ For this reason, the LSU is now more often used in the DNA barcoding of fungi.^9^ The amplified gene region is approximately 850 bp. Based on the observed variation in the LSU region, a phylogenetic tree can be readily established, see for instance Liu et al.^9^

Mushrooms are found in all possible ecosystems and the phylum contains many complex and highly advanced species that feed on predominantly dead organisms.^10^, ^11^ Fungi are categorized in different groups and identification of fungi serves many functions. The mushroom itself represents a fruit body of the fungus and the major part of the organism is generally found below the surface (the mycelium). The fruit body of many fungal species is made up of a stem, cap and gills of which the cap contains the conidia that play a role in the reproduction. Mushrooms can reproduce asexually (vegetative), which is the predominant form, and sexually. The asexual reproduction often manifests itself in a mass production and distribution of light small spores (derived from conidia). Fungi can spread rapidly through air and ventilation channels because of the spores. The sexual reproduction leads to a change in the DNA and allows fungi to adapt to a changing environment. An extensive review on fungi can be found elsewhere.^10^

Some mushrooms produce very potent toxic substances, posing major threats to human health or even leading to death,^11^, ^12^ and they differ significantly in ecology, pathogenicity and susceptibility to antifungal agents. A quick identification of mushrooms in case of poisoning can be of vital importance.^13^ Currently, biochemical tests are routinely used to determine the identity of different mushrooms. However, these techniques are rather time consuming. Recently, new methods have been developed to facilitate more rapid identification of poisonous mushrooms.^8^ Among them is the abovementioned DNA barcoding.

In this student project, performed by groups of three and four students over a time course of 6–8 weeks (full time), the identity and relationships between different mushroom species was investigated by DNA barcoding of the LSU gene. In the initial 2 weeks, students perform a field excursion collecting mushrooms and write a plan of approach. In these weeks, they also search for relevant literature. In the third, fourth and fifth weeks, they perform the actual extractions, DNA purifications and PCR amplifications. During these weeks, they also critically read a scientific article relevant for their work and present the paper in a journal club session. After the fifth week, the DNA is sent out for sequencing. In the sixth week, they interpret their results and create the phylogenetic tree. The seventh and eighth weeks are used for writing the report and presenting a poster. Students have individually evaluated the process, experimental work and outcome, especially with regards to their own professional development.

## 2 MATERIALS AND METHODS

### 2.1 Collection of materials

Mushrooms were collected in the third week of October under supervision of a professional mycologist in the Amsterdamse Bos. We collected Turkey tail (*Trametes versicolor*), magic button mushroom (*Agaricus geesterani*), sheathed woodtuft (*Kuehneromyces mutabilis*), grooved bonnet (*Mycena polygramma*), lumpy bracket (*Trametes gibbosa*), birch polypore (*Piptoporus betulinus*), bleeding fairy helmet (*Mycena haematopus*), shaggy parasol (*Chlorophyllum rhacodes*), and weeping widow mushroom (*Lacrymaria lacrymabunda*). Mushrooms collected were identified morphologically and photographed. A piece of the cap was removed wearing gloves and using a 70% ethanol‐sterilized knife and samples were stored in sterile tubes. The materials were stored at −20°C until further analysis. *Agaricus bisporus* was obtained from a local supermarket.

### 2.2 DNA isolation

After collection, DNA was isolated from the fresh caps using the DNeasy plant tissue kit (Qiagen). Isolations were carried out as described by the supplier. In brief, 100 mg of each mushroom cap was measured and homogenized with a pestle. A total of 400 μl AP1 buffer and 4 μl RNase A were added to a 1.5 ml Eppendorf tube. The samples were vortexed and incubated under rotation for 10 min at 65°C. After the incubation, 130 μl of P3 buffer was added and samples were incubated on ice for 5 min. Subsequently, samples were centrifuged for 5 min at 20,000*g* and supernatants were transferred to a Qiashredder spin column (Qiagen). Samples were then recentrifuged at 20,000*g* for 2 min. The flow‐through was transferred to a clean tube. A total of 1.5 volumes of buffer AW1 was added and 650 μl of this mixture was transferred to a DNeasy Mini spin column (Qiagen). Further isolation was exactly as described by the manufacturer.

### 2.3 PCR amplification

PCR amplification using a C1000 instrument (Bio‐Rad) of the LSU rRNA was performed on the samples using forward primer LR0R 5′‐ACCCGCTGAACTTAAGC and reverse primer LR5 5′‐TCCTGAGGGAAACTTCG.^4^ Before amplification, concentration of the DNA was determined using a NanoDrop spectrophotometer (Thermofisher). In all amplification reactions 5 ng of mushroom DNA was used. PCR included a 6 min denaturation step, 35 cycles including 30 s denaturation (95°C), 40 s annealing (50°C) and 60 s extension (72°C). An additional 8 min extension step after cycling was included. After PCR, samples were loaded onto a 1.5% agarose gel containing Sybr Safe (Thermofisher). The gel was run for 40 min at 100 Volt. The fragments were analyzed using a Gel Doc EZ system (Bio‐Rad).

### 2.4 DNA extractions and purification

DNA fragments were isolated from gel using the X‐Tracta gel extractor (Promega) and fragment weights were measured. A total of 3 volumes of QG buffer was pipetted to 1 volume of gel (100 mg equals 100 μl). The samples were subsequently incubated at 50°C for 10 min and vortexed every 2 min to dissolve the gel. A total of 800 μl of the mixture was transferred to a Qiaquick spin column (Qiagen). After centrifugation, 0.5 ml QG buffer was added and spin columns were centrifuged for 1 min. Further washing and elution, using PE and EB buffers, were exactly as indicated by the supplier. Samples were stored at −20°C until further use.

### 2.5 Sequencing

The purified DNA samples were Sanger sequenced at BaseClear (the Netherlands). The required concentration of the samples was 15 ng/μl per 100 bp and required volumes were 20 μl (consisting of 6 ng/μl DNA and 25 pmol primer LR0R or primer LR5).

### 2.6 Alignment and phylogenetic analysis

The raw trace files were edited and the consensus sequences were aligned using the built‐in Geneious Global alignment algorithm (free end gaps and 65% similarity settings, Geneious 11.1.5, Biomatters), with *Calocera viscosa* as outgroup. The sequenced region included a sequence of maximally 975 bp, starting on position 616 and ending on position 1591 in NCBI Nucleotide file MT014000 (this is an example file for LSU DNA sequence analysis based on the primers used in this study). The alignment was based on a 600 bp consensus region. A phylogenetic tree was created using Geneious software applying maximum parsimony (using the PAUP 4.0 plug‐in) to calculate the genetic relationships using *C. viscosa* as outgroup. All obtained trees were resampled by the bootstrap method by performing 1,000 iterations to assess statistical confidence of the clades.

## 3 RESULTS AND DISCUSSION

### 3.1 Collection of materials

**FIGURE 1:**
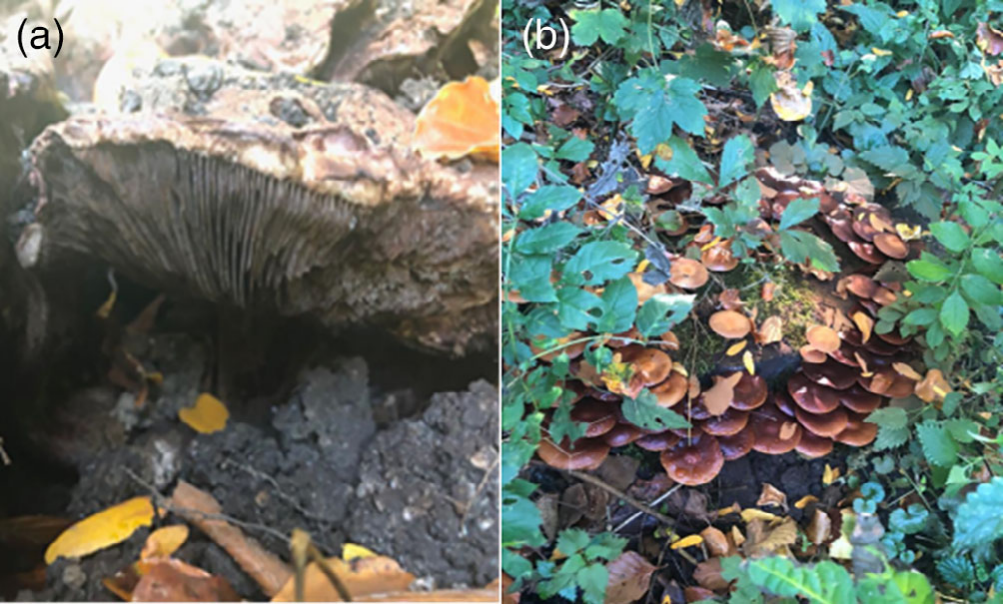
Fruiting bodies of two mushroom species collected and analyzed. (a) *Agaricus geesterani*; (b) *Kuehneromyces mutabilis* [Color figure can be viewed at wileyonlinelibrary.com]

Before fresh mushrooms were collected in the field, all species were photographed by the students. Representative pictures of the fruiting bodies of *A. geesterani* and *K. mutabilis*, photographed from below, are depicted in Figure 1.

### 3.2 DNA isolation

The DNA isolation steps were monitored by quantitation and by gel electrophoresis. DNA concentrations ranged between 7 ng/μl and 51 ng/μl (data shown in Supporting Information Data S1). Absorbance 260/280 ratios were variable, ranging between 1.4 and 5.3, indicating that the purity of the DNA of the samples was not very high. Since these samples were taken in the field, we cannot exclude some soil to have contaminated the sampling. DNA yields were sufficient for subsequent amplification though. The DNA isolation from *A. bisporus* was rather high compared to the other isolations. This is the only mushroom which was not covered by soil particles as it was retrieved from a local supermarket and the more hygienic condition might be the reason for the higher DNA yield. As an alternative to NanoDrop quantitation, DNA may be measured using a Qubit instrument (Thermofisher) or by standard UV spectroscopy.

### 3.3 PCR amplification

**FIGURE 2:**
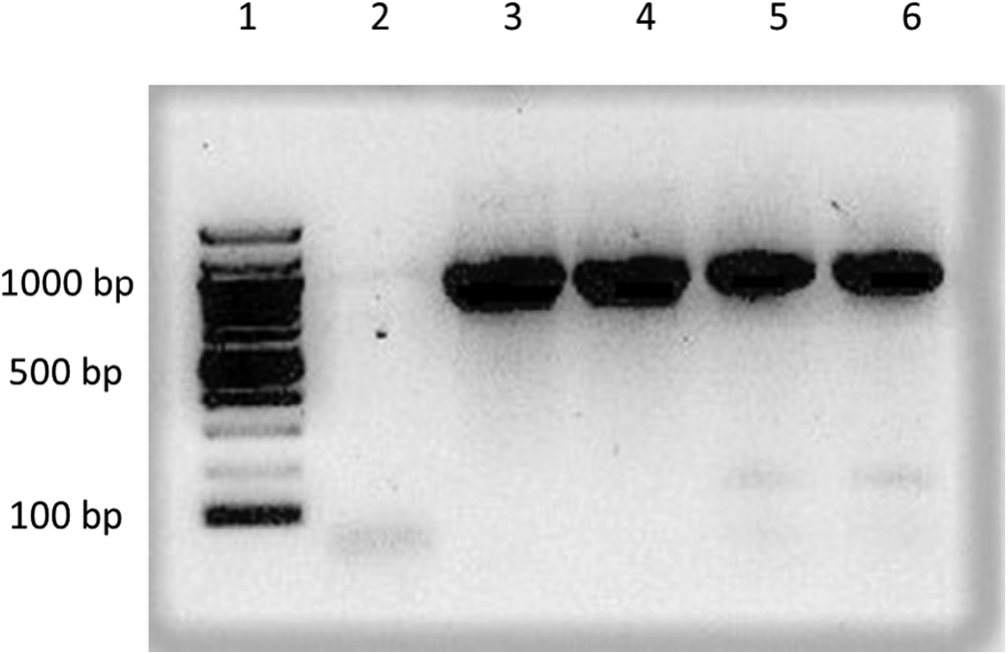
Isolated representative amplicons of the large subunit gene from four different mushrooms. Amplicons shown represent *Mycena haematopus*, *Chlorophyllum rhacodes*, *Lacrymaria lacrymabunda* and *Piptoporus betulinus*, Lanes 3, 4, 5 and 6, respectively. The predicted product size is approximately 850 bp. Lane 1 contains a 100 bp size ladder and Lane 2 shows a nontemplate control in which a primer dimer fragment is present

The purpose of the PCR was to amplify the LSU gene. After the PCR reactions were completed, samples were analyzed by agarose gel electrophoresis (see Figure 2 for four representative amplicons). Surprisingly, taking the purity of the samples into consideration, all samples yielded the expected DNA fragment of approximately 800 bp, demonstrating the robustness of the method. A PCR amplification without input DNA did not yield a product, indicating that no contamination had occurred during the mushroom DNA isolation and amplification steps.

### 3.4 DNA extraction and purification

Fragments obtained were isolated from the gel using the X‐tracta Gel extractor and purified using a Qiaquick Gel Extraction kit. This step can be omitted, since unique PCR products can be sequenced directly, however the retrieved sequences may be of lower quality. As an alternative to avoid lower quality DNA, an amplicon clean‐up may be done using a PCR product clean‐up kit. A NanoDrop analysis was performed to determine the DNA concentration and purity of the samples. For Sanger sequencing, the optimal DNA concentration should be at least 15 ng per 100 bp. The results of the NanoDrop analysis are shown in Supporting Information Data S1, demonstrating that samples had a purity level around 1.8 and slightly variable sample concentrations. During the experimental work, documenting was a learning objective and at a certain point, DNA concentrations were not well registered and had to be remeasured. The issue of sufficient documentation is a general challenge and students reflected on this (see under “reflections”).

### 3.5 Sequencing

Sanger sequencing was done by BaseClear (Leiden, the Netherlands) and in‐house at the Leiden Centre of Applied Biosciences using an ABI Prism 3100 instrument (Thermofisher). Full DNA sequences can be found in the Supporting Information Data S1. Sequences were of very high quality and could be read for at least 600 bp. Both forward and reverse sequencing was performed in case of doubt.

### 3.6 Alignment

**FIGURE 3:**
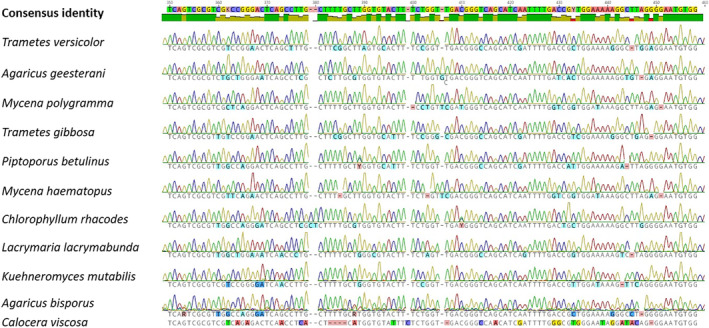
Geneious alignment of mushroom large subunit (LSU) sequences. Alignment was based on a 520 bp part of the LSU region. A variable part of the consensus region is shown [Color figure can be viewed at wileyonlinelibrary.com]

DNA sequences were analyzed in Geneious Prime. Based on the trimmed sequences, an alignment was made using the built‐in Geneious aligner. A minor part of the alignment demonstrating considerable variability is shown in Figure 3. Sanger sequencing yielded sequences up to 800 bp and alignment was based on a part in the LSU region spanning 520 bp (for more detailed coordinates of the aligned region: see for instance NCBI Nucleotide file MT014000, positions 684–1204). In case of ambiguities or difficult base calling, DNA samples were resequenced.

As an alternative to Geneious, CLUSTAL omega, MEGA software or Unipro UGENE can be used for alignment. These tools are freely available on the internet.

### 3.7 Phylogenetic analysis

**FIGURE 4:**
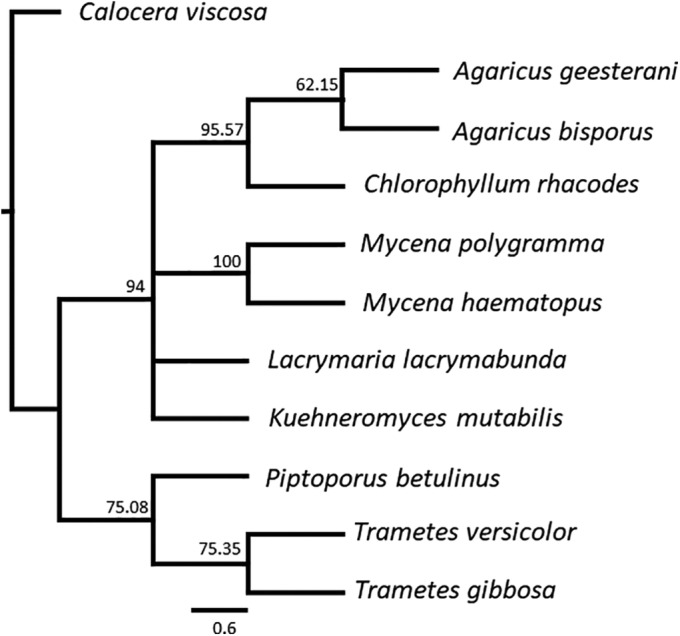
Consensus phylogenetic tree of the different mushroom species. *Calocera viscosa* was used as outgroup

In order to verify morphologically based identifications and determine phylogenetic relationships, DNA sequences were analyzed phylogenetically in Geneious. The yellow stagshorn (*C. viscosa*) functioned as an outgroup (NCBI accession number MH867841). It belongs to a different class (Dacrymycetes), that shares a common ancestor with the class of the Agaricomycetes^14^, ^15^, ^16^ to which all orders belong from which we sampled the different mushrooms analyzed in this study. The resulting phylogenetic tree is shown in Figure 4, numbers indicated at the branches represent the support percentages for the nodes in the tree. The topology supplied the students with information on the phylogenetic relationships based on outgroup and ingroup comparison.

First, most DNA sequences generated matched with data in the reference database of identical species, confirming the identifications based on morphology. Second, the two *Mycena* species ended up in the same clade, which was also the case for the two *Agaricus* and *Trametes* species. Based on morphology, the two *Mycena* species are quite alike and they can easily be recognized by the color of the stalk. The relatedness was clearly confirmed by the DNA analysis. The same holds true for the two *Trametes* species, which can be rapidly identified in the field and their morphological difference as well as relatedness were supported by the DNA analysis. The analysis also confirmed the differences between the different mushroom orders. We analyzed mushrooms from the orders Polyporales and Agaricales. Polyporales are characterized by the presence of pores in the fruiting body when observed from below. Agaricales have clear lamellae (gills) in the fruiting body.^10^ *P. betulinus*, *T. versicolor*, and *T. gibbosa* all belong to the order of Polyporales, which was confirmed by the DNA analyses. The difference between the *Trametes* species and *P. betulinus*, which can be clearly observed in the field, was supported by their DNA sequence divergence as these species did not end up in the same clade. It is known from literature that these species belong to different families (*P. betulinus* belongs to the Fomitopsidaceae and the *Trametes* species belong to the Polyporaceae^16^). The two *Agaricus* species, the two *Mycena* species, as well as *L. lacrymabunda*, *K. mutabilis*, and *C. rhacodes* all belong to the order of the Agaricales. The rare *A. geesterani*, which has a limited geographical distribution, was also analyzed in this project. Literature searching confirmed that the classification of this species is disputed and it may very well belong to a different genus than *Agaricus*, called *Allopsalliota*.^17^ The close relatedness of *A. geesterani* to *C. rhacodes* was shown using other tree building algorithms in which *A. geesterani* clustered with *C. rhacodes* (data not shown). As expected, *C. viscosa*, the only mushroom belonging to the class of the Dacrymycetes, was found to be more distantly related to all other species in our study. The results are in line with published phylogenies on mushrooms.^14^, ^15^, ^16^ Students can easily find more information on the evolutionary relationships of mushrooms on the web using for instance the Tree of Life Web Project portal^15^ or other resources.

We conclude that students can rapidly verify morphologically based identifications of mushrooms collected in the field by DNA barcoding in an educational project. In addition, they can assess phylogenetic relationships of the various mushroom species collected using this methodology in a 6–8 week student project setting.

### 3.8 Data deposition

The DNA sequence of *A. geesterani*, not previously deposited in the UNITE database, was uploaded to the server (UDB0778789|Iv01, unite.ut.ee).^18^

### 3.9 Reflections

In the described student project, the seven students that chose this project were introduced to the concept of DNA barcoding as a powerful tool to study biodiversity. After the project, all students were individually asked to reflect on the research done. In addition, a short evaluation with the group was done. The most important aspects are presented here.

All students in this group emphasized the importance of teamwork. While most of the practical work in our studies is done in a classic form, this work was done in a project‐form, in which students cooperate, divide tasks and design a good planning. All students indicated the importance of the teamwork, adequate communication, accuracy and sufficient documentation as a crucial factor for a successful project. One of them stated that they needed each other to do the work and come to good conclusions. After finishing the work, the students had a good view on how to perform a complete line of molecular research. The project‐based work also reinforced the interest in biology in general and students indicated that they learned more than by doing a classical practical. Students were furthermore surprised to see that molecular biology on mushrooms, unexpectedly, was straightforward and a good example for biomolecular work in general. Finally, all students indicated that solving research questions independently and being coached in their work by a mentor was a highly motivating part of their studies. The work resulted in students being proud of the results obtained and of the scientific poster based on their work. In this way students demonstrated true ownership of their work.

## 4 CONCLUSION

The DNA barcoding project on mushrooms described in this article is a relatively easy, yet challenging research project that can be performed by students with a basic background in molecular biology. The costs of the project are relatively low as students make use of general lab equipment and general molecular biology reagents. The field work, as well as the applied bioinformatics part form added values to the work. Mushrooms used in the study are common ones that can be found in different continents; basically all mushrooms of which LSU sequences have been deposited in NCBI GenBank, UNITE or any other database can be used in the project. Literature and database searches as well as the laboratory experiments, provide students with a complete and simple research line in which they learn to cooperate, understand the importance of documenting, being accurate and reporting scientific results.

## CONFLICT OF INTEREST

The authors declare that there are no conflicts of interest.

## AUTHOR CONTRIBUTIONS

Ivo R. Horn designed the study and wrote the manuscript together with Barbara Gravendeel. Peter A. Verleg, Nafiesa Z. Ibrahim, Khadiedjah Soeleman, Floris van Kampen, Mia O. Ruesen, Naïsha M. Reulen, and Roderick Bakker were the students involved in the project. Henk Breij was the mycologist involved in the fieldwork and morphological identification of the mushrooms.

## Supporting information


**Data S1**. DNA concentrations and *A*_260/280_ ratios with taxonomic information and retrieved LSU sequences of the investigated mushrooms. DNA concentrations and *A*_260/280_ ratios after DNA isolation and after gel extraction are shown and full retrieved sequence information is presented.
