# Supplementary material for: Mushroom DNA barcoding project: Sequencing a segment of the 28S rRNA gene
Source: Biochem Mol Biol Educ. 2020 Jun 25;48(4):404–10. doi: 10.1002/bmb.21388 (PMC7497104; doi:10.1002/bmb.21388)
Supplement: Supplementary file 1 — Data S1. DNA concentrations and A 260/280 ratios with taxonomic information and retrieved LSU sequences of the investigated mushrooms. DNA concentrations and A 260/280 ratios after DNA isolation and after gel extraction are shown and full retrieved sequence information is presented. [file BMB-48-404-s001.docx]

**Supportive information S1**

**DNA concentrations and A_260/280_ ratios with taxonomic information of the investigated mushrooms.**

|  |  |  |  |  |
| --- | --- | --- | --- | --- |
| Species | DNA concentration | A_260/280_ | DNA concentration | A_260/280_ |
|  | after DNA isolation | after DNA isolation | after gel extraction | after gel isolation |
|  | ng/µl |  | ng/µl |  |
| *Agaricus bisporus*  (J.E. Lange) Pilát | 51.1 | 4.8 | 10.6 | 2.6 |
| *Trametes versicolor*  (L.) Pilát | 8 | 5.3 | 8.0 | 2.7 |
| *Agaricus geesterani*  Bas and Heinem. | 12.1 | < 0 | 12.0 | 2.0 |
| *Kuehneromyces mutabilis*  (Schaeff) Singer & A.H. Sm. | 31.6 | < 0 | 30.3 | 1.9 |
| *Mycena polygramma*  (Bull.) Gray | 7.3 | 2.4 | 8.0 | 1.3 |
| *Trametes gibbosa*  (Pers.) Fr. | 7.3 | 1.3 | 7.0 | 1.6 |
| *Piptoporus betulinus*  (Bull.) P. Karst. | 15 | < 0 | 14 | 2.0 |
| *Mycena haematopus*  (Pers.) P. Kumm. | 13.3 | 1.6 | 13.0 | 1.7 |
| *Chlorophyllum rhacodes*  (Vittad.) Vellinga | 18.4 | 1.4 | 19.0 | 2.1 |
| *Lacrymaria lacrymabunda*  (Bull.) Gray | 12.7 | 1.4 | 13.0 | 1.7 |
|  |  |  |  |  |
|  |  |  |  |  |
|  |  |  |  |  |

**Retrieved LSU sequences of the investigated mushrooms.**

*Piptoporus betulinus*

TTCCCCTAGTAACTGCGAGTGAAGCGGGAAAAGCTCAAATTTAAAATCTGGCGGTCTTATGGCCGTCCGAGTTGTAGTCTGGAGAAGTGCTTTCCGCGCTGGACCGTGTACAAGTCTCTTGGAACAGAGCGTCATAGAGGGTGAGAATCCCGTCTTTGACACGGACTACCAGTGCTTTGTGATGCGCTCTCAAAGAGTCGAGTTGTTTGGGAATGCAGCTCAAAATGGGTGGTAAATTCCATCTAAAGCTAAATATTGGCGAGAGACCGATAGCGAACAAGTACCGTGAGGGAAAGATGAAAAGCACTTTGGAAAGAGAGTTAAACAGTACGTGAAATTGCTGAAAGGGAAACGCTTGAAGTCAGTCGCGTTGGCCAGGACTCAGCCTTGCTTTTGCTYGGTGCATTTTCTGGTTGACGGGCCAGCATCGATTTTGACCATTGGAAAAAGATTAGGGGAATGTGGCACCTTCGGGTGTGTTATAGCCCTTAGTCACATACAGTGGTTGGGATCGAGGACCGCAGCACGCCT

*Agaricus bisporus*

TTCCCCTAGTAACTGCGAGTGAAGCGGGAAAAGCTCAAATTTAAAATCTGGCGGTCTTTGGCCGTCCGAGTTGTAATCTAGAGAAGTAATGCCCGTGCTGGACCGTGTATAAGTCTCCTGGGATGGGGCGTCATAGAGGGTGAGAATCCCGTCTTTGACACGGACTACCAGTGCATTGTGGTATGCTCTCAAAGAGTCGAGTTGTTTGGGAATGCAGCTCAAAATGGGTGGTAAATTCCATCTAAAGCTAAATATTGGCGAGAGACCGATAGCGAACAAGTACCGTGAGGGAAAGATGAAAAGAACTTTGGAAAGAGAGTTAAACAGTACGTGAAATTGCTGAAAGGGAAACGCTTGAAGTCARTCGCGTTGGCCAGGGATCAGCCTTGCTTTTGCRTGGTGTACTTTCTGGTTGACGGGTCAGCATCAATTTTGACCGCTGGAAAAGGGCCTGGGGAATGTGGCAGCTTCKGCTGTGTTATAGCCCCTGGTCSCATACWGTGGWTGGGATTGARGAACTCASCACGCCGCAA

*Trametes versicolor*

TTCCCCTAGTAACTGCGAGTGAAGCGGGAAAAGCTCAAATTTAAAATCTGGCGGTCTTTGGCCGTCCGAGTTGTAGTCTGGAGAAGCGTCTTCCGCGTTGGACCGTGTACAAGTCTCTTGGAACAGAGCGTCATAGAGGGTGAGAATCCCGTCTTTGACACGGACTACCAATGCTTTGTGATGCGCTCTCAAAGAGTCGCGTTGTTTGGGAATGCAGCGCAAAATGGGAGGTGAATTCCTTCTAAAGCTAAATATTGGCGAGAGACCGATAGCGAACAAGTACCGTGAGGGAAAGATGAAAAGCACTTTGGAAAGAGAGTTAAACAGTACGTGAAATTGCTAAAAGGGAAACGCTTGAAGTCAGTCGCGTCGTCCGGAACTCAGCTTTGCTTCGGCTTAGTGCACTTTCCGGTTGACGGGCCAGCATCGATTTTGACCGCTGGAAAAGGGCTGGAGGAATGTGGCACCTTCGGGTGTGTTATAGCCTTCAGTCGCATACAGCGGTTGGGATCGAGGAACGCAGCGCGCCTTATGGC

*Mycena haematopus*

TTCCCCTAGTAACTGCGAGTGAAGCGGGAAAAGCTCAAATTTAAAATCTGGCGGTCCYTGTGGCCGTCCGAGTTGTAATTTAGAGAAGYGTTATCCGYGCTAGACCGTGTACAAGTCTCCTGGAATGGAGCGTCATAGAGGGTGAGAATCCCGTCTTTGACACGGACTGCTAGGRCATTGTGATGCRCTCTCAAAGAGTCGAGTTGTTTGGGAATGCAGCTCAAAATGGGTGGTAAATTCCATCTAAAGCTAAATATTGAGGAGAGACCGATAGCGAACAAGTACCGTGAGGGAAAGATGAAAAGAACTTTGGAAAGAGAGTTAAACAGTACGTGAAATTGCTGAAAGGGAAACGCTTGAAGTCAGTCGCGTCGTTCAGAACTCAGCCTTGCTTTGCTTGGTGTACTTTCTGTTCGACGGGTCAGCATCAATTTTGGTCGGTGGATAAAGGCTTAGAGAATGTGGCATCCTCGGATGTGTTATAGCTCTGGGTCGCATACATCGGCTGGGATTGAGGAACTCAGCACGCC

*Chlorophyllum rhacodes*

TTCCCCTAGTAACTGCGAGTGAAGCGGGAAAAGCTCAAATTTAAAATCTGGCGGTCTTTGGCTGCCCGAGTTGTAATCTAGAGAAGTAATGCCCGCGCTGGACCGTGTACAAGTCTCCTGGAATGGAGCGTCATAGAGGGTGAGAATCCCGTCTCTGACACGGACTACCAGGGCTTTGTGGTATGCTCTCAAAGAGTCGAGTTGTTTGGGAATGCAGCTCAAAATGGGTGGTAAATTCCATCTAAAGCTAAATATTGGCGAGAGACCGATAGCGAACAAGTACCGTGAGGGAAAGATGAAAAGAACTTTGGAAAGAGAGTTAAACAGTACGTGAAATTGCTGAAAGGGAAACGCTTGAAGTCAGTCGCGTTGGCCAGGGATCAGCCTCGCTCTTTTGCGTGGTGTACTTTCTGGTTGAYGGGTCAGCATCAATTTTGACTGCTGGAAAAAGGCTTGGGGGAATGTGGCAGCTTCGGCTGTGTTATAGCCCCTGGTCACATACAGCGGTTGGGATTGAGGAACTCAGCACGCCGCA

*Calocera viscosa* (outgroup)

TAGTAACTGCGAGTGAAGCGGGAAAAGCTCAAATTTGTAATCCGTCCCCCGACGGAGTTGTAATCTAGAGAAGTGTTTTCGGCCGTTGTCTCGGATAAGTCCCCTGAAACAGGGCGTCATAGAGGGTAACAATCCCGTCCTTACCGAGGTCGCAATGTCTATGTGATACACTCTCGAAGAGTCGAGTTGTTTGGGAATGCAGCTCAAAATGGGTGGTAAATTCCATCTAAAGCTAAATATTGGCGAGAGACCGATAGCGAACAAGTACCGTGAGGGAAAGATGAAAAGCACTTTGGAAAGAGAGTTAAACAGTACGTGAAATTGCTAAAAGGGAAGCGCTTGAAGTCAGTCGCGTCGTCAGAGACTCAACCTCACTCATGGTGTATTTCTCTGGTGACGGGCCAACATCGATTTTGGGCGTGGGAATAGGATACAGGGAATGTGGCAGCCTCGGCTGTGTTATAGCCCTGCAATCGTAAACCACGCCTGGGATCGAGGACCGCGGCTCTGCCTA

*Kuehneromyces mutabilis*

TTCCCCTAGTAACTGCGAGTGAAGCGGGAAAAGCTCAAATTTAAAATCTGGCGGTCTTTGGCTGTCCGAGTTGTAATCTAGAGAAGTGTTATCCGCGCTGGACCGTGTACAAGTCTCCTGGAATGGAGCGTCATAGAGGGTGAGAATCCCGTCTTTGACACGGACTGCCAGTGCTTTGTGATGCGCTCTCAAAGAGTCGAGTTGTTTGGGAATGCAGCTCAAAATGGGTGGTAAATTCCATCTAAAGCTAAATATTGGCGAGAGACCGATAGCGAACAAGTACCGTGAGGGAAAGATGAAAAGAACTTTGGAAAGAGAGTTAAACAGTACGTGAAATTGTTGAAAGGGAAACGCTTGAAGTCAGTCGCGTCGTCCGGGGATCAACCTTGCTTTTGCTGGGTGTACTTTCCGGTTGACGGGTCAGCATCAATTTTGACCGTTGGATAAAGTTCAGGGGAATGTGGCATCTTCGGATGTGTTATAGCCCTTGGTCGCATACAACGATTGGGATTGAGGAACTCAGCACGCCG

*Mycena polygramma*

TCCCCTAGTAACTGCGAGTGAAGCGGGAAAAGCTCAAATTTAAAATCTGGCGGTCCTTGTGGCCGTCCGAGTTGTAATTTAGAGAAGTGTTATCCGCGCTGGACCGTGTACAAGTCTCCTGGAATGGAGCGTCATAGAGGGTGAGAATCCCGTCTTTGACACGGACTGCCAGGGCATTGTGATGCGCTCTCGAAGAGTCGAGTTGTTTGGGAATGCAGCTCAAAATGGGTGGTAAATTCCATCTAAAGCTAAATATTGGGGAGAGACCGATAGCGAACAAGTACCGTGAGGGAAAGATGAAAAGAACTTTGGAAAGAGAGTTAAACAGTACGTGAAATTGCTGAAAGGGAAACGCTTGAAGTCAGTCGCGTCGCTCAGGACTCAGCCTTGCTTTTGCTTGGTGTACTTCCTGTTCGATGGGTCAGCATCAATTTTGGTCGGTGGATAAAGGCTTAGAGAATGTGGCATCTTCGGGTGTGTTATAGCTCTGGGTCGCATACATCGGCTGGGATTGAGGAACTCAGCACGCCGCAAGGCC

*Agaricus geesterani*

TTCCCCTAGTAACTGCGAGTGAAGCGGGAAAAGCTCAAATTTAAAATCTGGCGGTCTTTGGCCGTCCGAGTTGTAATCTAGAGAAGTAATGCCCGTGCTGGACCGTATACAAGTCTCCTGGGATGGAGCATCATGGAGGGTGAGAATCCCGTACCTGATACGGACTACCAGTACATTGTGGTATGCTTTCAACGAGTCGAGTTGTTTGGGAATGCAGCTCAAAATGGGTGGTAAATTCCATCTAAAGCTAAATATTGGCGAGAGACCGATAGCGAACAAGTACCGTGAGGGAAAGATGAAAAGAACTTTGGAAAGAGAGTTAAACAGTACGTGAAATTGCTGAAAGGGAAACGCTTGAAGTCAGTCGCGTCTGCTGGGAATCAGCCTCGCTCTTGCGTGGTGTACTTTCTGGTGTGACGGGTCAGCATCAATTTTGATCACTGGAAAAAGGTGTGAGGAATGTGGCAGCTTCGGCTGTGTTATAGACTCGCGTCGTATACAGTGGTTGGGATTGAGGAACGCAGCACGCCGC

*Lacrymaria lacrymabunda*

TTCCCCCTAGTWAACTGCGAGTGAAGCGGGAAAAGCTCAAATTTAAAATCTGGTGGTCTTTGGCCATCCGAGTTGTAATCTAGAGAAGTGTTACCCGCGCCGGACCGTGTATAAGTCTCCTGGAATGGAGCGTCATAGAGGGTGAGAATCCCGTCTTTGACACGGACTACCGGGGCTTTTGTGGTGCGCTCTCAAAGAGTCGAGTTGTTTGGGAATGCAGCTCAAAATGGGTGGTAAATTCCATCTAAAGCTAAATATTGGCGAGAGACCGATAGCGAACAAGTACCGTGAGGGAAAGATGAAAAGAACTTTGGAAAGAGAGTTAAACAGTACGTGAAATTGCTGAAAGGGAAACGCTTGAAGTCAGTCGCGTTGGCTGGAAATCAACCCTGCTTTTGCTGGGCGTACTTTCTAGTTGACGGGCCAGCATCAGTTTTGACCGGTGGAAAAAGTCTAGGGGAATGTGGCATCTTCGGATGTGTTATAGCCCTTGGTCGTATACATCGGTTGGGACTGAGGAACTCAGCACGCCGCAAGGCCGG

Trametes gibbosa

TTCCCCTAGTAACTGCGAGTGAAGCGGGAAAAGCTCAAATTTAAAATCTGGCGGTCTTTGGCCGTCCGAGTTGTAGTCTGGAGAAGTGCTTTCCGCGCTGGACCGTGTACAAGTCTCTTGGAACAGAGCGTCATAGAGGGTGAGAATCCCGTCTTTGACATGGACTACCAGTGCTTTGTGATGCGCTCTCAAAGAGTCGAGTTGTTTGGGAATGCAGCTCAAAATGGGTGGTGAATTCCATCTAAAGCTAAATATTGGCGAGAGACCGATAGCGAACAAGTACCGTGAGGGAAAGATGAAAAGCACTTTGGAAAGAGAGTTAAACAGTACGTGAAATTGCTGAAAGGGAAACGCTTGAAGTCAGTCGCGTTGTCCGGAACTCAGCCTTGCTTCGGCTTGGTGCATTTTCCGGGCGACGGGCCAGCATCGATTTTGACCGTCGGAAAAGGGCTGAGGGAATGTGGCACCTTCGGGTGTGTTATAGCCTTCAGTCGCATACGGCGGTTGGGATCGAGGAACGCAGCAC
